# Metabolic differences in *MSTN* and *FGF5* dual-gene edited sheep muscle cells during myogenesis

**DOI:** 10.1186/s12864-024-10494-w

**Published:** 2024-06-26

**Authors:** Mingming Chen, Yan Li, Xueling Xu, Shuqi Wang, Zhimei Liu, Shiyu Qi, Dandan Si, Zhuo Man, Shoulong Deng, Guoshi Liu, Yue Zhao, Kun Yu, Zhengxing Lian

**Affiliations:** 1 Beijing Key Laboratory for Animal Genetic Improvement, National Engineering Laboratory for Animal Breeding, Key Laboratory of Animal Genetics and Breeding of the Ministry of Agriculture, College of Animal Science and Technology, China Agricultural University, Beijing, 100193 China; 2 Laboratory Animal Center of the Academy of Military Medical Sciences, Beijing, 100071 China; 3 SCIEX China, Beijing, 100015 China; 4 National Center of Technology Innovation for animal model, NHC Key Laboratory of Human Disease Comparative Medicine, Institute of Laboratory Animal Sciences, Chinese Academy of Medical Sciences and Comparative Medicine Center, Peking Union Medical College, Beijing, China

**Keywords:** *MSTN*, *FGF5*, Sheep, Skeketal muscle cells, Metabolomics

## Abstract

Dynamic metabolic reprogramming occurs at different stages of myogenesis and contributes to the fate determination of skeletal muscle satellite cells (MuSCs). Accumulating evidence suggests that mutations in myostatin (MSTN) have a vital role in regulating muscle energy metabolism. Here, we explored the metabolic reprogramming in MuSCs and myotube cells in *MSTN* and *FGF5* dual-gene edited sheep models prepared previously, and also focused on the metabolic alterations during myogenic differentiation of MuSCs. Our study revealed that the pathways of nucleotide metabolism, pantothenate and CoA biosynthesis were weakened, while the unsaturated fatty acids biosynthesis were strengthened during myogenic differentiation of sheep MuSCs. The *MSTN* and *FGF5* dual-gene editing mainly inhibited nucleotide metabolism and biosynthesis of unsaturated fatty acids in sheep MuSCs, reduced the number of lipid droplets in per satellite cell, and promoted the pentose phosphate pathway, and the interconversion of pentose and glucuronate. The *MSTN* and *FGF5* dual-gene editing also resulted in the inhibition of nucleotide metabolism and TCA cycle pathway in differentiated myotube cells. The differential metabolites we identified can be characterized as biomarkers of different cellular states, and providing a new reference for *MSTN* and *FGF5* dual-gene editing in regulation of muscle development. It may also provide a reference for the development of muscle regeneration drugs targeting biomarkers.

**Supplementary Information:**

The online version contains supplementary material available at 10.1186/s12864-024-10494-w.

## Introduction

Myogenesis is a multi-step biological process in which skeletal muscle satellite cells (MuSCs) are activated, proliferated, differentiated, migrated, and fused to form multinucleate myotubes. It is driven by a variety of regulators, such as paired box family (Pax3/7), myogenic regulatory factors (Myf5, MyoD, Myogenin, and MRF4/6) and myogenic enhancer 2 (MEF2) family proteins [1–3], which together regulate the muscle-specific gene expression and control myogenesis and skeletal muscle development.

Accumulating evidence indicates that MuSCs undergo dynamic metabolic reprogramming at distinct stages of the myogenic differentiation [4–6]. The quiescent MuSCs requirement and are barely rely on glycolysis, and mainly rely on mitochondrial ATP production through oxidative phosphorylation and fatty acid β-oxidation [7]. Compared to the quiescent state, activated MuSCs exhibit a metabolic transition from the oxidation of fatty acid to higher rates of glycolysis, with upregulated levels of glycolysis [7]. After entering terminal differentiation, myoblasts must synthesize ATP at an elevated rate to maintain intracellular reorganization that accompanies differentiation, thus differentiated myoblasts mainly depend on oxidative phosphorylation to provide energy [8–10].

Myostatin (MSTN) is derived from the superfamily of transforming growth factor-β (TGF-β), which inhibits skeletal muscle growth and development in an autocrine and paracrine manner [11]. Fibroblast growth factor 5 (FGF5) is a branch of fibroblast growth factor family and has a negative efficacy on hair growth and development [12]. It has been shown that mutations in the *MSTN* gene regulate muscle energy metabolism levels and lead to a transformation of muscle fiber types. For example, *MSTN* knockout increases the proportion of fast muscle fibers leading to muscle hypertrophy in mice [13]. Proteins related to the fast muscle fiber phenotype in Belgian blue cattle are up-regulated, suggesting that mutations in the *MSTN* gene may lead to a massive hyperplasia of fast glycolytic muscle fibers [14].

In our previous study, we prepared *MSTN* and *FGF5* dual-gene edited sheep, which showed the phenotype of muscle fiber hyperplasia [15]. Here, we use this model to explore the differences in metabolic levels of sheep MuSCs and myotube cells by *MSTN* and *FGF5* dual-gene edited, so as to provide a reference for *MSTN* and *FGF5* dual-gene editing-mediated muscle fiber hyperplasia.

## Materials and methods

### Cell isolation, culture, and induction of differentiation

As we previously reported isolation methods, MuSCs were isolated from 3-month-old male embryos of Dorper sheep, which were derived from WT or *MSTN* and *FGF5* dual-gene edited heterozygotes (MF^+/−^) [16, 17]. After resuspension of the resuscitated MuSCs in DMEM/F12 growth medium (GM) supplemented with 20% fetal bovine serum (FBS) and 1% penicillin/streptomycin (P/S) solution, and then incubated cells at 37℃ in a 5% CO_2_ incubator. Until the cells achieved 70% confluence, the medium was then replaced with differentiation medium (DM) supplemented with 2% horse serum (HS) and 1% P/S solution in DMEM high glucose to induce MuSCs myogenic differentiation for 2 days (DM2).

### Preparation of metabolomics samples

A total of 1 × 10^7^ skeletal muscle cells were collected by 0.25% trypsin digestion, resuspended with 1 mL pre-cooled methanol: acetonitrile: water (2:2:1, v/v), and the cells were broken by repeated freeze-thaw with liquid nitrogen three times. Then, the samples were rested at -20 °C for 1 h, centrifuged at 12,000 rpm at 4 °C for 15 min, the supernatant was lyophilized in a vacuum freeze dryer. Subsequently, each sample was dissolved in 100 µL acetonitrile: water (1:1, v/v). The samples were vortexed for 30 s, ultrasonicated in ice for 5 min, and centrifuged at 12,000 rpm for 15 min at 4 °C. The supernatant was taken and analyzed by mass spectrometry in the injection vial.

### LC-MS/MS data collection

The SCIEX X500R liquid chromatography-quadrupole tandem time-of-flight mass spectrometer was employed for data collection. The chromatographic conditions were as follows: A mobile phase: H_2_O (containing 0.1% formic acid); B mobile phase: acetonitrile (containing 0.1% formic acid); Column temperature: 40 °C; Maximum pressure resistance: 19,000 psi; Injection volume: 5 µL; Flow rate: 200 µL/min. The conditions of mass spectrometry were: ESI+& ESI: positive and negative ion acquisition; Ion source temperature: 550 °C; MS primary mass number range: 60–1300 m/z; MS/MS secondary fragment acquisition mode: 15 MS/MS, 50–1300 m/z; Secondary collision energy: 30 ± 15 eV.

### Metabolomics data analysis

The xcms package of R software was used for peak extraction and filtering. The SCIEX OS database and MetDNA2 (http://metdna.zhulab.cn/) were used for metabolite recognition. MetaboAnalyst 5.0 (https://www.metaboanalyst.ca/) was used for data filtering, standardization, and pathway analysis. The t-test was used for inter group difference analysis, and *P*-value < 0.05, Fold Change (FC) > 1.5 and < 1/1.5 as screening criteria for differential metabolites. Meanwhile, orthogonal partial least squares discriminant analysis (OPLS-DA) was implemented by SIMCA (v14.1), where the PSL-DA model was tested for 200 permutations, and the variable important in projection (VIP) was calculated. Finally, VIP > 1 was used as the threshold for further screening of metabolites and was used as the final identified differential metabolite.

### The staining of lipid droplets and cytoskeleton

Sheep MuSCs were cultured to 50% confluence and stabilized with 4% paraformaldehyde. Then, cells were treated with 0.5% Triton X-100 for 10 min, followed by incubation with phalloidin (4 U/mL, Solarbio Life Sciences, Beijing) for 20 min in the dark. Next, cells were stained by 5 µM BODIPY 493/503 (Cayman Chemical, Michigan) at 37 °C in the dark for 15 min. Finally, the nuclei were stained with DAPI, and anti-fluorescence attenuation sealing agent was used for sealing. The images were captured by super-resolution laser confocal microscopy (Nikon Corporation, Japan).

### Statistical analysis

At least four bio-replicates were arranged for each group, all data were exhibited as the mean ± SEM. The two-tailed *Student’s t*-test was employed for statistical analysis of differences between groups, with *P* < 0.05 as statistically significant. *P < 0.05, **P < 0.01 and ***P < 0.001.

## Results

### LC-MS/MS data quality control of non-targeted metabolomics

**Fig. 1:**
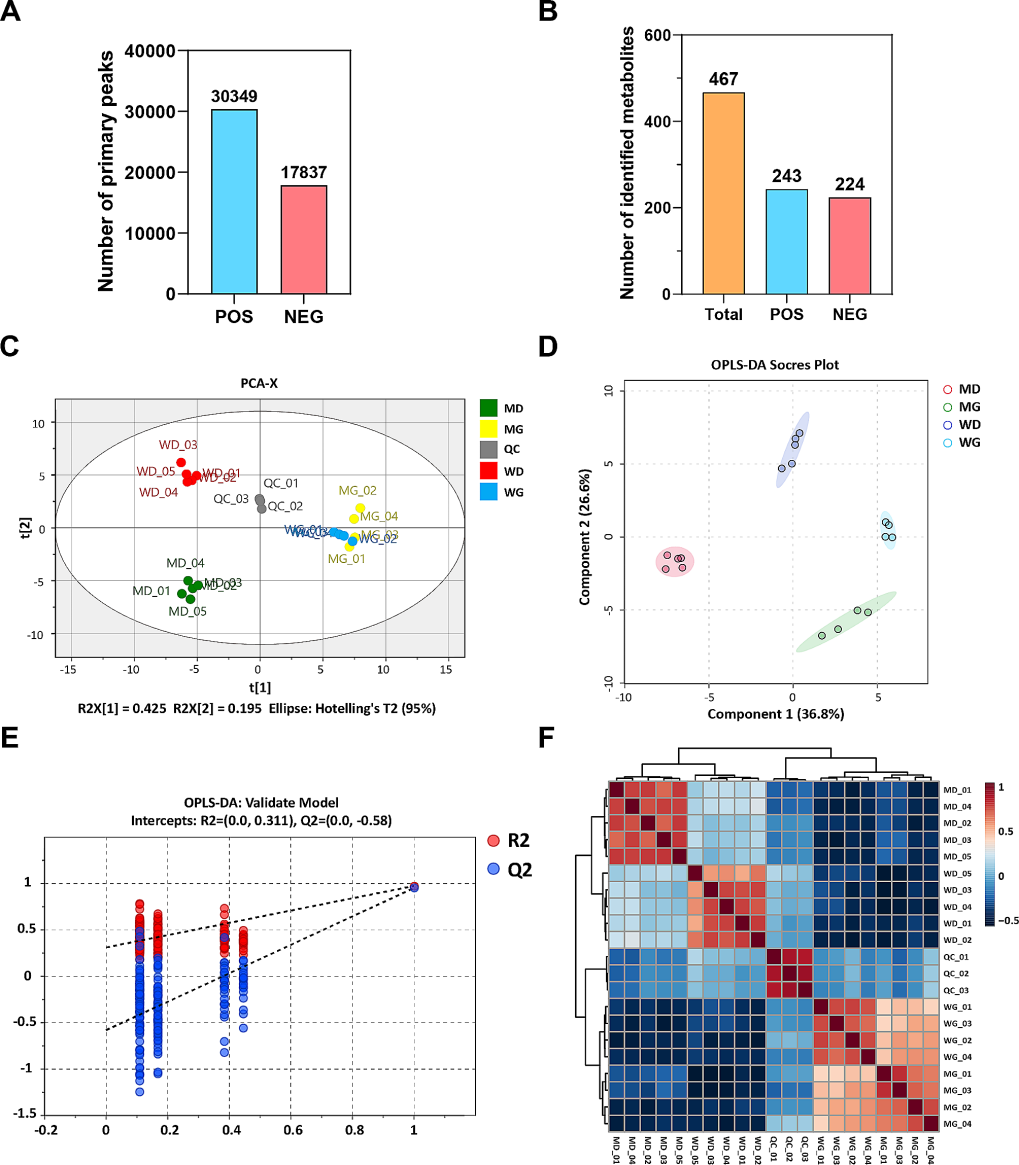
LC-MS/MS data quality control of non-targeted metabolomics. (**A**) The number of peaks extracted by the primary mass spectrometry in the positive and negative ion mode. (**B**) The number of identified metabolites in the positive and negative ion mode. (**C**) Unsupervised PCA analysis of QC samples. (**D**) OPLS-DA scores plot. (**E**) OPLS-DA model replacement test. (**F**) Sample correlation analysis. WG: WT sheep skeletal muscle satellite cells at GM; WD: WT sheep myotube cells at DM2; MG: MF^+/−^ sheep skeletal muscle satellite cells at GM; MD: MF^+/−^ sheep myotube cells at DM2

We previously prepared *MSTN* and *FGF5* dual-gene edited sheep by injecting Cas9 mRNA and sgRNAs into transferable embryos, which highlighted a “dual-muscle” phenotype and myofiber hyperplasia [15, 18]. At the cellular level, *MSTN* and *FGF5* dual-gene editing promotes the proliferation and inhibits myogenic differentiation of sheep MuSCs [15]. In this study, we focused on the metabolic differences in *MSTN* and *FGF5* dual-gene edited sheep muscle cells during myogenesis. Peak recognition, filtration and alignment were performed on the raw mass spectrometry data, and a total of 30,349 peaks were extracted from the primary mass spectrometry in positive ion mode, and 17,837 peaks were extracted from the primary mass spectrometry in negative ion mode (Fig. 1A, Supplementary Table 1). Qualitative analysis of the primary and secondary mass spectra of these peaks identified a total of 467 metabolites, of which 243 metabolites were identified in positive ion mode and 224 metabolites were identified in negative ion mode (Fig. 1B, Supplementary Table 2).

All metabolites were subjected to RSD% statistics and metabolites with RSD > 30% were removed, and metabolite abundance data were log-transformed and pareto-scaled. To evaluate the stability of QC samples, unsupervised PCA analysis was performed and the results showed that all QC samples were densely distributed, with samples clustered with each other within each group and more discrete between groups, indicating high data quality and significant differences between samples (Fig. 1C). Furthermore, OPLS-DA was performed on all samples by SIMCA software, and the results showed excellent intra-sample group repeatability and large inter-sample group dispersion and discrimination (Fig. 1D). To estimate whether the OPLS-DA supervised model was overfitted, the model was tested with 200 permutations, the results demonstrated that the Q2 value was − 0.58 and the intersection of the regression line of Q2 with the y-axis was less than 0, indicating that the OPLS-DA model was not overfitted (Fig. 1E). Pearson’s correlation analysis indicated a high degree of similarity within the sample group, suggesting low intra-sample error and high reproducibility (Fig. 1F). In summary, the LC-MS/MS mass spectrometry data has high quality and can be used for subsequent analysis.

### Metabolic differences in sheep MuSCs during myogenic differentiation

**Fig. 2:**
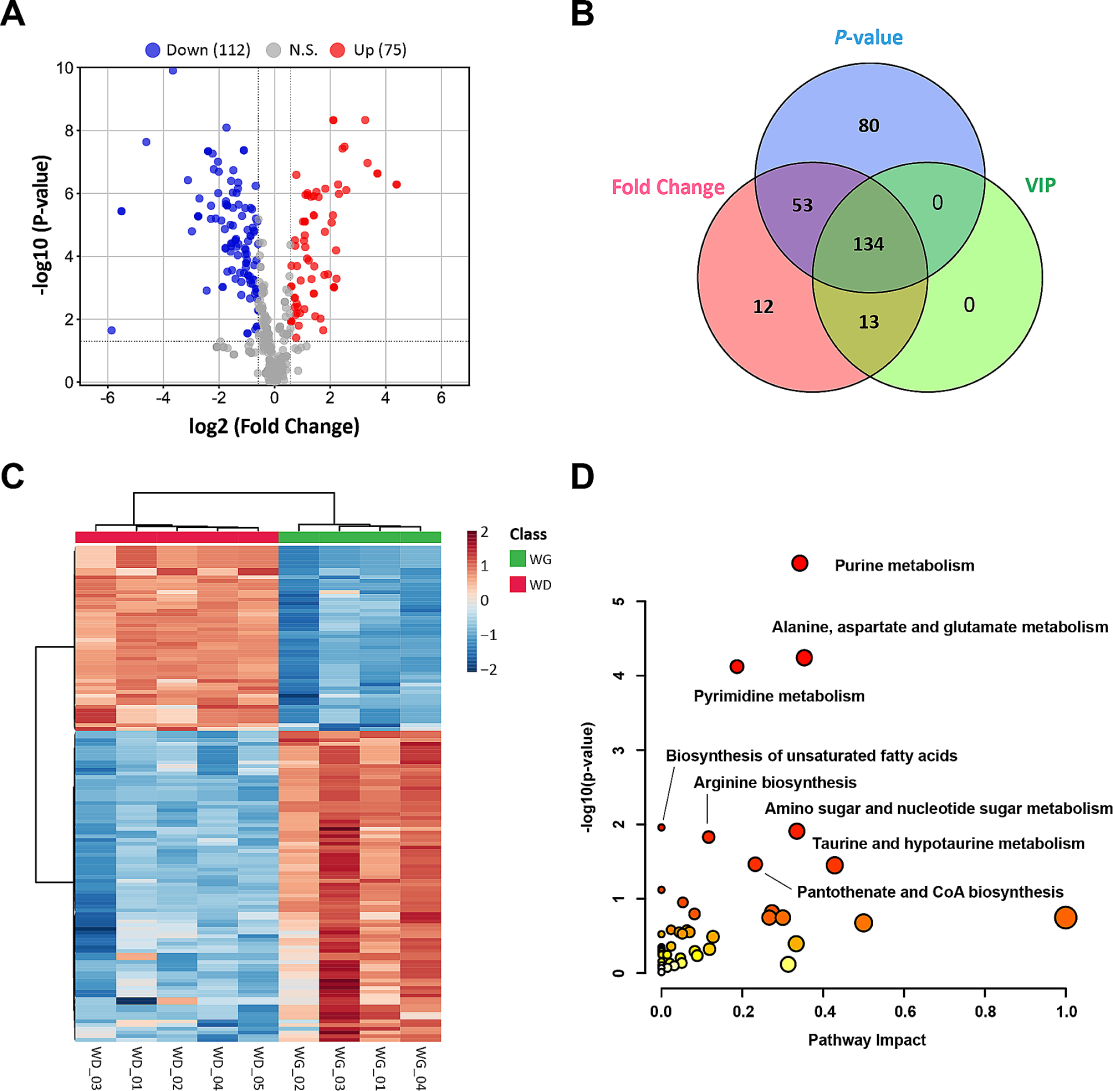
Identification of differential metabolites and pathway analysis during myogenic differentiation of sheep skeletal muscle satellite cells. (**A**) Volcano plot of differential metabolites. (**B**) *P*-value, Fold Change and VIP Veen diagrams. (**C**) Heat map of differential metabolite clustering. (**D**) Differential metabolite pathway analysis. WG: WT sheep skeletal muscle satellite cells at GM; WD: WT sheep myotube cells at DM2

To investigate the differences in metabolic levels during myogenic differentiation of sheep MuSCs and their effects on proliferation and myogenic differentiation, we performed a differential metabolites analysis of MuSCs and myotube cells. A total of 187 differential metabolites were recognized by *P*-value < 0.05, FC > 1.5 and < 1/1.5 (Fig. 2A). To characterize the contribution of metabolites in sample differentiation, VIP > 1 was used as a threshold for further screening, and ultimately identified 134 differential metabolites (Fig. 2B, Supplementary Table 3). The heat map of differential metabolite clustering indicated that the samples are clustered into two categories, with significant differences between sample groups (Fig. 2C). To identify the biochemical metabolic pathways that different metabolites may be involved, pathway analysis of the differential metabolites was performed. Results demonstrated that the differential metabolites of sheep MuSCs and myotube cells were significantly (*P* < 0.05) enriched in purine metabolism, the metabolism of alanine, aspartate and glutamate, pyrimidine metabolism, the metabolism of amino sugar and nucleotide sugar, taurine and hypotaurine metabolism, pantothenate and CoA biosynthesis, the biosynthesis of unsaturated fatty acids, and arginine biosynthesis (Fig. 2D).

**Fig. 3:**
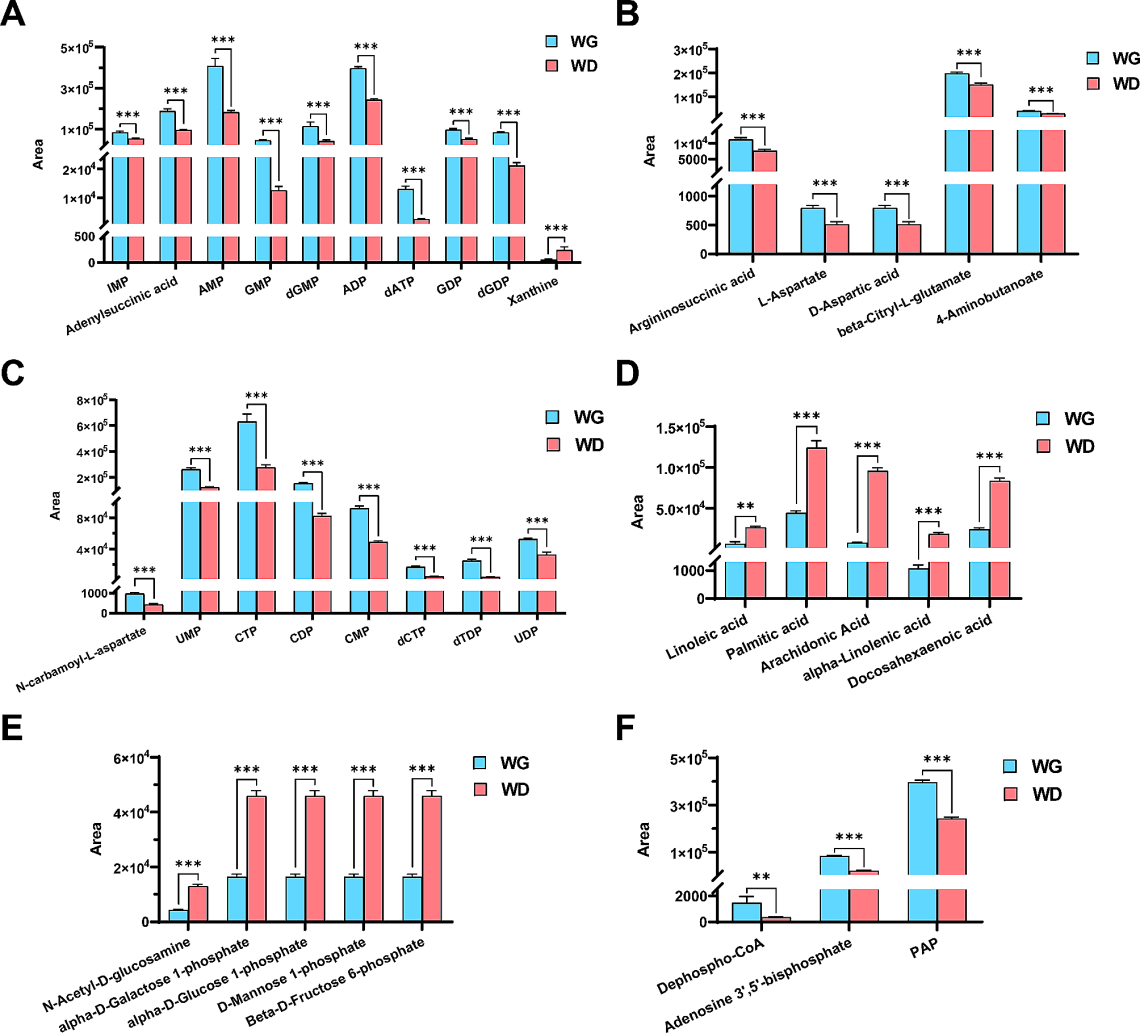
Expression profile of differential metabolites during myogenic differentiation of sheep skeletal muscle satellite cells. (**A**) Differential metabolite expression profiles related to purine metabolism. (**B**) Differential metabolite expression profiles related to alanine, aspartate and glutamate metabolism. (**C**) Differential metabolite expression profiles related to pyrimidine metabolism. (**D**) Differential metabolite expression profiles related to biosynthesis of unsaturated fatty acids. (**E**) Differential metabolite expression profiles related to amino sugar and nucleotide sugar metabolism. (**F**) Differential metabolite expression profiles related to pantothenate and CoA biosynthesis. WG: WT sheep skeletal muscle satellite cells at GM; WD: WT sheep myotube cells at DM2

To characterize and dissect the physiological significance of biochemical metabolic pathways during myogenic differentiation of sheep MuSCs, the differential metabolite expression profiles of MuSCs and myotube cells were analyzed. Compared with MuSCs, adenylsuccinic acid, inosine monophosphate (IMP), guanosine monophosphate (GMP), adenosine monophosphate (AMP), adenosine diphosphate (ADP), guanosine diphosphate (GDP), deoxyguanosine monophosphate (dGMP), deoxyadenosine triphosphate (dATP) and deoxyguanosine diphosphate (dGDP) were all significantly (*P* < 0.001) decreased in the purine metabolism pathway of myotube cells, while xnathine level was significant (*P* < 0.001) increased (Fig. 3A). The arginosuccinic acid, L-aspartate, D-aspartic acid, β-citryl-L-glutamate, and 4-aminobutanoate in alanine, aspartate and glutamate metabolism pathways were all significantly (*P* < 0.001) reduced (Fig. 3B). The N-carbamyl-L-aspartate, uridine diphosphate (UDP), uridine monophosphate (UMP), cytidine diphosphate (CDP), cytidine monophosphate (CMP), cytidine triphosphate (CTP), deoxycytidine triphosphate (dCTP) and deoxythymidine diphosphate (dTDP), which are related to pyrimidine metabolism, were also significantly (*P* < 0.001) decreased in myotube cells (Fig. 3C). In addition, the unsaturated fatty acids, such as linoleic acid, palmitic acid, arachidonic acid, α-linolenic acid and docosahexaenoic acid were markedly (*P* < 0.01) up-regulated in myotube cells (Fig. 3D), and N-acetyl-glucosamine, galactose-1-phosphate, glucose-1-phosphate, fructose-6-phosphate, and mannose-1-phosphate of amino sugar and nucleotide sugar metabolic pathways were also dramatically (*P* < 0.01) up-regulated (Fig. 3E). The dephospho-CoA, adenosine 3’,5’-bisphosphate (P2A), and adenosine 3’,5’-diphosphate (PAP) in pantothenate and CoA biosynthesis were remarkably (*P* < 0.01) decreased in myotube cells (Fig. 3F).

In summary, the nucleotide metabolism, pantothenate and CoA biosynthesis pathways were weakened, while unsaturated fatty acid biosynthesis pathways was strengthened during myogenic differentiation.

### Metabolic differences in MSTN and FGF5 dual-gene edited MuSCs in sheep

**Fig. 4:**
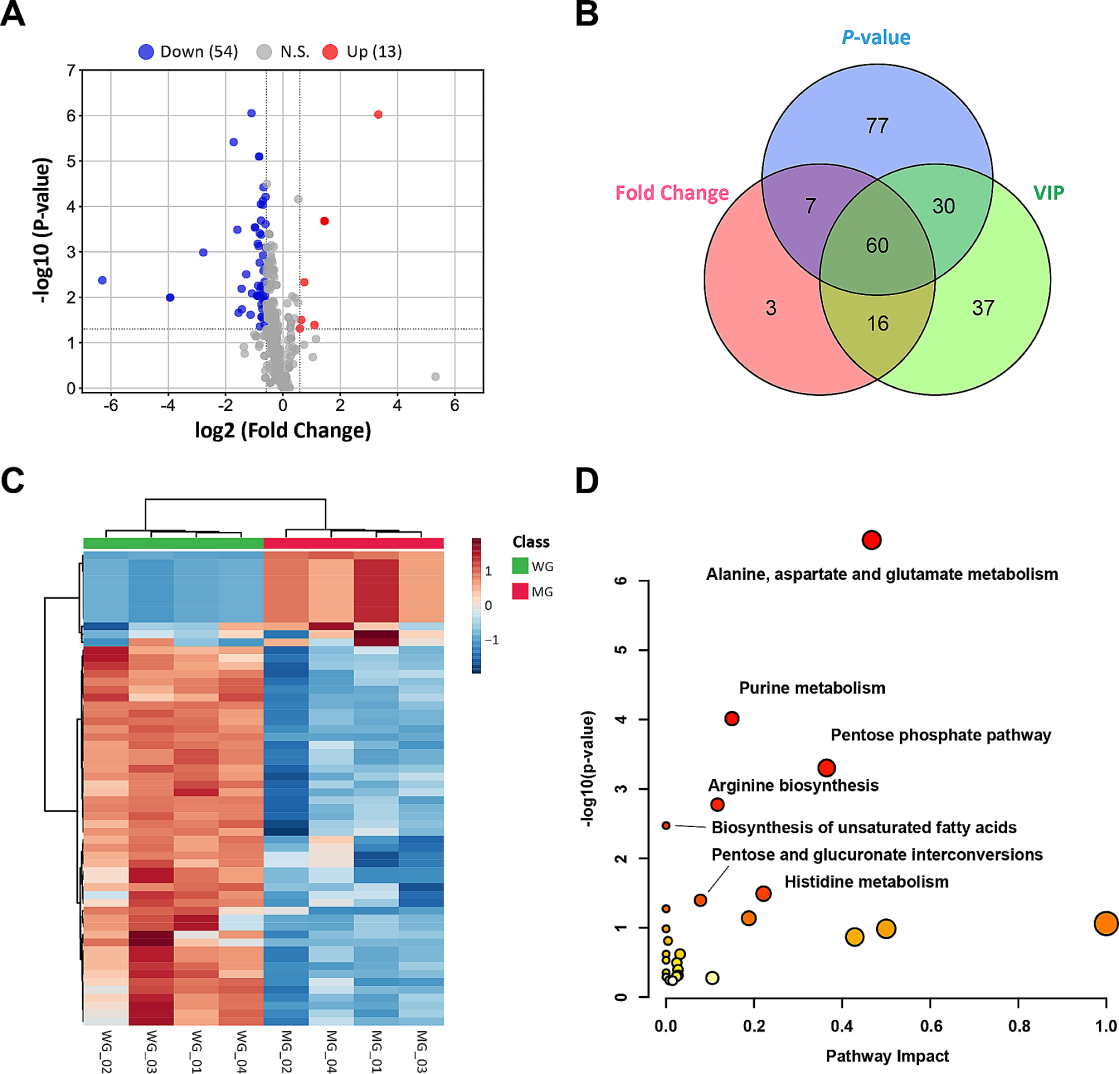
Identification of differential metabolites and pathway analysis of WT and MF^+/-^ sheep skeletal muscle satellite cells at GM. (**A**) Volcano plot of differential metabolites. (**B**) *P*-value, Fold Change and VIP Veen diagrams. (**C**) Heat map of differential metabolite clustering. (**D**) Differential metabolite pathway analysis. WG: WT sheep skeletal muscle satellite cells at GM; MG: MF^+/−^ sheep skeletal muscle satellite cells at GM

To investigate the effect of *MSTN* and *FGF5* dual-gene editing on the metabolic level of sheep MuSCs, we performed a differential metabolites analysis between WT and MF^+/−^ sheep MuSCs. A total of 67 differential metabolites were recognized by *P*-value < 0.05, FC > 1.5 and < 1/1.5 (Fig. 4A). To characterize the contribution of metabolites in sample differentiation, VIP > 1 was used as a threshold for further screening, and ultimately identified 60 differential metabolites (Fig. 4B, Supplementary Table 4). The heat map of differential metabolite clustering indicated that the samples are divided into two categories, with significant differences between sample groups (Fig. 4C). The results of differential metabolite pathway analysis showed that the differential metabolites of WT and MF^+/−^ sheep MuSCs were significantly (*P* < 0.05) enriched in alanine, aspartate and glutamate metabolism, purine metabolism, pentose phosphate pathway, arginine biosynthesis, histidine metabolism, unsaturated fatty acids biosynthesis, pentose and glucuronate interconversions (Fig. 4D).

**Fig. 5:**
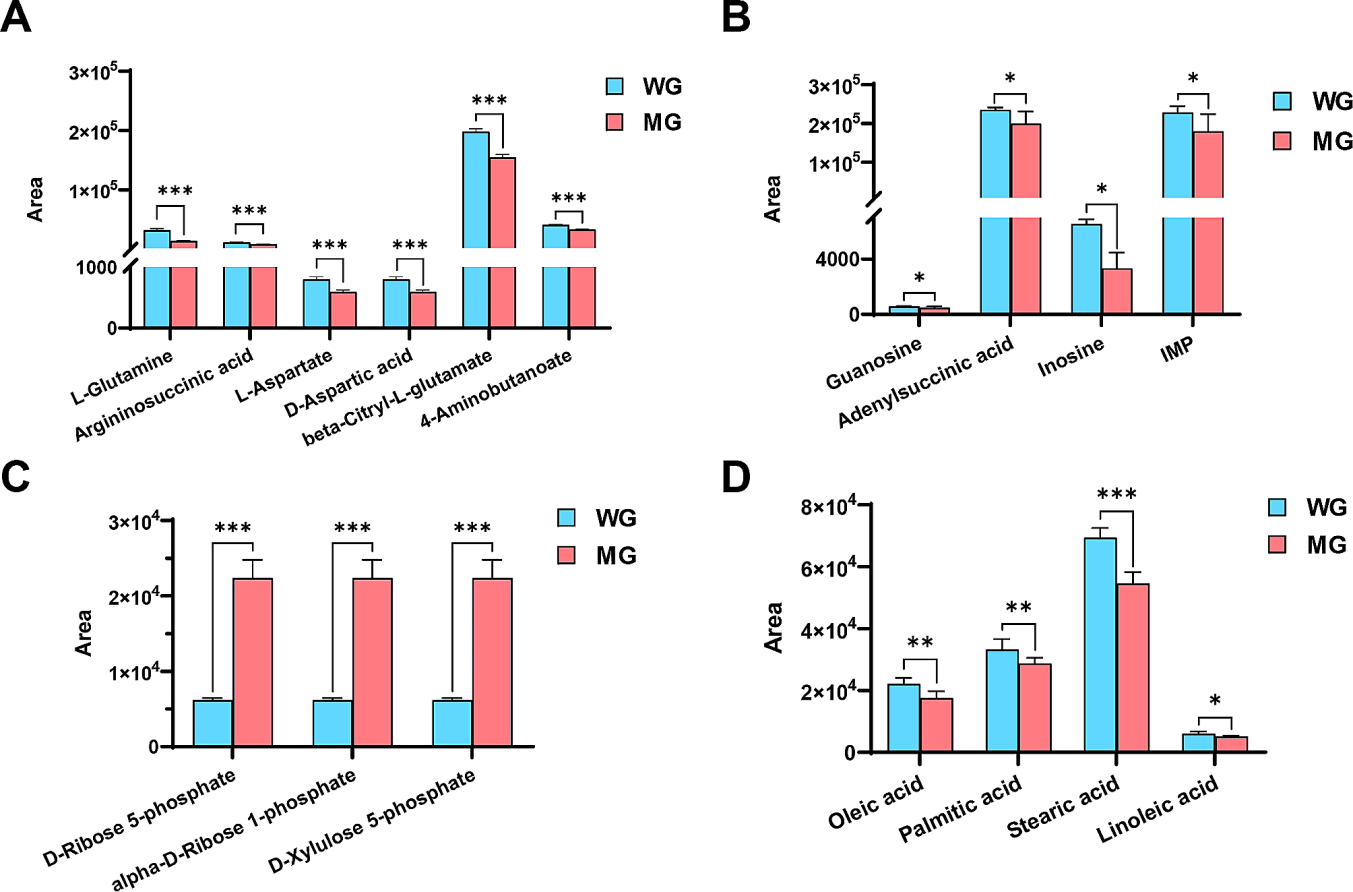
Expression profile of differential metabolites between WT and MF^+/-^ sheep skeletal muscle satellite cells at GM. (**A**) Differential metabolite expression profiles related to alanine, aspartate and glutamate metabolism. (**B**) Differential metabolite expression profiles related to purine metabolism. (**C**) Differential metabolite expression profiles related to pentose phosphate pathway, pentose and glucuronate interconversions. (**D**) Differential metabolite expression profiles related to biosynthesis of unsaturated fatty acids. WG: WT sheep skeletal muscle satellite cells at GM; MG: MF^+/−^ sheep skeletal muscle satellite cells at GM

Further differential metabolite expression profiling revealed that the L-glutamine, argininosuccinic acid, L-aspartate, D-aspartic acid, β-citryl-L-glutamate, and 4-aminobutyrate in the alanine, aspartate and glutamate metabolic pathways were dramatically significant (*P* < 0.001) diminished in MF^+/−^ MuSCs compared to WT cells (Fig. 5A), which was paralleled by the metabolic level in WT myotube cells. Similarly, the levels of guanosine, inosine, adenylsuccinic acid, and IMP were also markedly (*P* < 0.05) diminished in the purine metabolism pathway in MF^+/−^ MuSCs (Fig. 5B). In addition, levels of ribose-5-phosphate, ribose-1-phosphate and xylulose-5-phosphate involved in the pentose phosphate pathway, pentose and glucuronate interconversions were all significantly (*P* < 0.001) enhanced in MF^+/−^ MuSCs compared to WT cells (Fig. 5C).

**Fig. 6:**
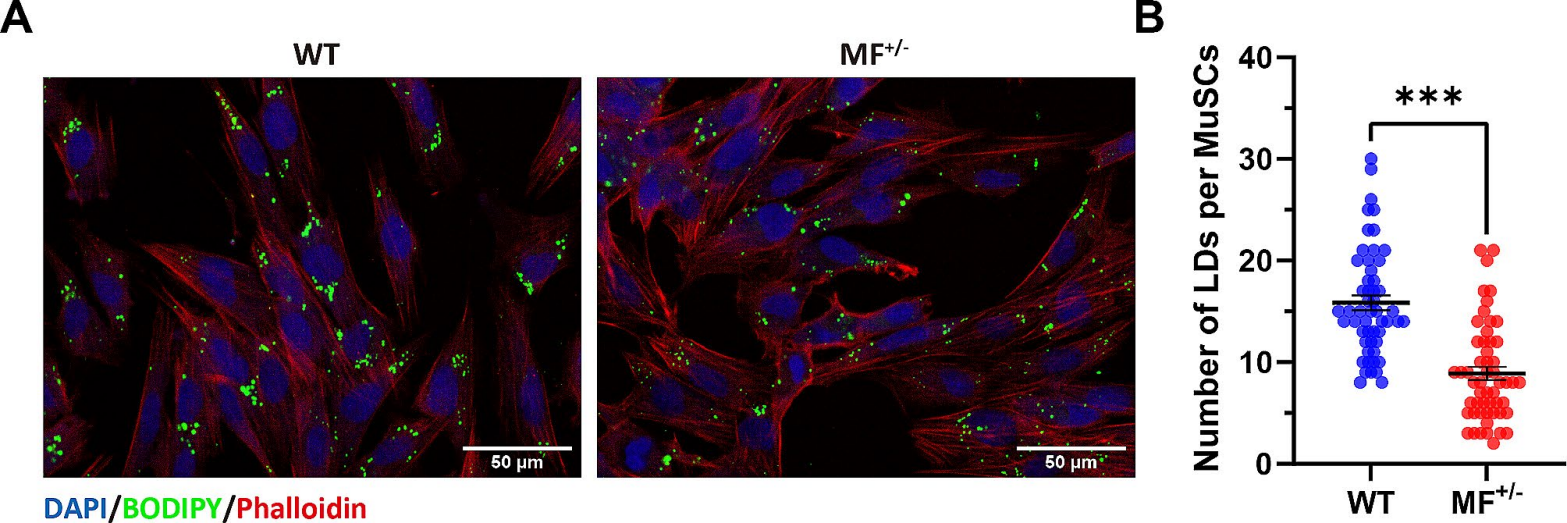
Effects of*MSTN* and *FGF5* dual-gene editing on lipid droplets (LDs) of sheep skeletal muscle satellite cells. (**A**) The LDs staining on WT and MF^+/−^ sheep skeletal muscle satellite cells. DAPI (blue) stained the nucleus, phalloidin (red) stained the cytoskeleton, and BODIPY (green) labeled LDs. Scale bar 50 μm. (**B**) The number of lipid droplets in per skeletal muscle satellite cell

Compared with WT MuSCs, the levels of oleic acid, linoleic acid, stearic acid, and palmitic acid in MF^+/−^ cells were significantly (*P* < 0.05 ) decreased (Fig. 5D), which was contrary to the metabolic level in myotube cells after myogenic differentiation. Further, we found that the number of lipid droplets in per MF^+/−^ skeletal muscle satellite cell was dramatically (*P* < 0.001) reduced compared to WT cells (Fig. 6A-B), suggesting that the limited biosynthesis of unsaturated fatty acids may affect the lipid droplet formation and ultimately reduce the energy intake of MF^+/−^ sheep MuSCs.

### Metabolic differences in MSTN and FGF5 dual-gene edited sheep myotube cells

**Fig. 7:**
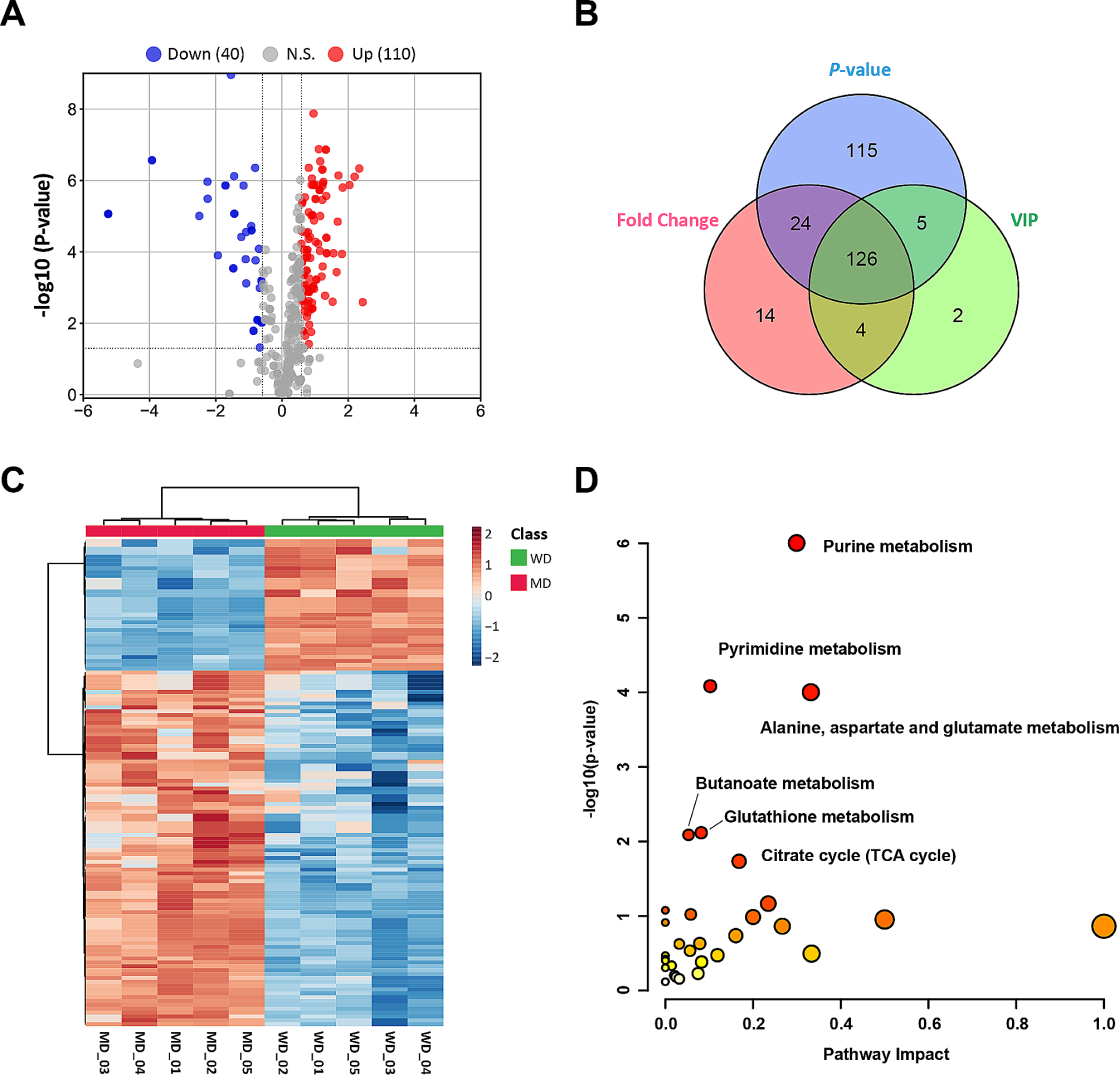
Identification of differential metabolites and pathway analysis of WT and MF^+/-^ sheep skeletal muscle satellite cells at DM2. (**A**) Volcano plot of differential metabolites. (**B**) *P*-value, Fold Change and VIP Veen diagrams. (**C**) Heat map of differential metabolite clustering. (**D**) Differential metabolite pathway analysis. WD: WT sheep myotube cells at DM2; MF^+/−^ sheep myotube cells at DM2

To explore the effect of *MSTN* and *FGF5* dual-gene editing on the metabolic level of sheep myotube cells, we performed a differential metabolites analysis between WT and MF^+/−^ sheep myotube cells. A total of 150 differential metabolites were recognized by *P*-value < 0.05, FC > 1.5 and < 1/1.5 (Fig. 7A). To characterize the contribution of metabolites in sample differentiation, VIP > 1 was used as a threshold for further screening, and ultimately identified 126 differential metabolites (Fig. 7B, Supplementary Table 5). The heat map of differential metabolite clustering indicated that the samples are divided into two categories, with significant differences between sample groups (Fig. 7C). The results of differential metabolite pathway analysis showed that the differential metabolites of WT and MF^+/−^ sheep myotube cells were significantly (*P* < 0.05) enriched in purine metabolism, pyrimidine metabolism, alanine, aspartate and glutamate metabolism, butanoate metabolism, glutathione metabolism, and TCA cycle (Fig. 7D).

**Fig. 8:**
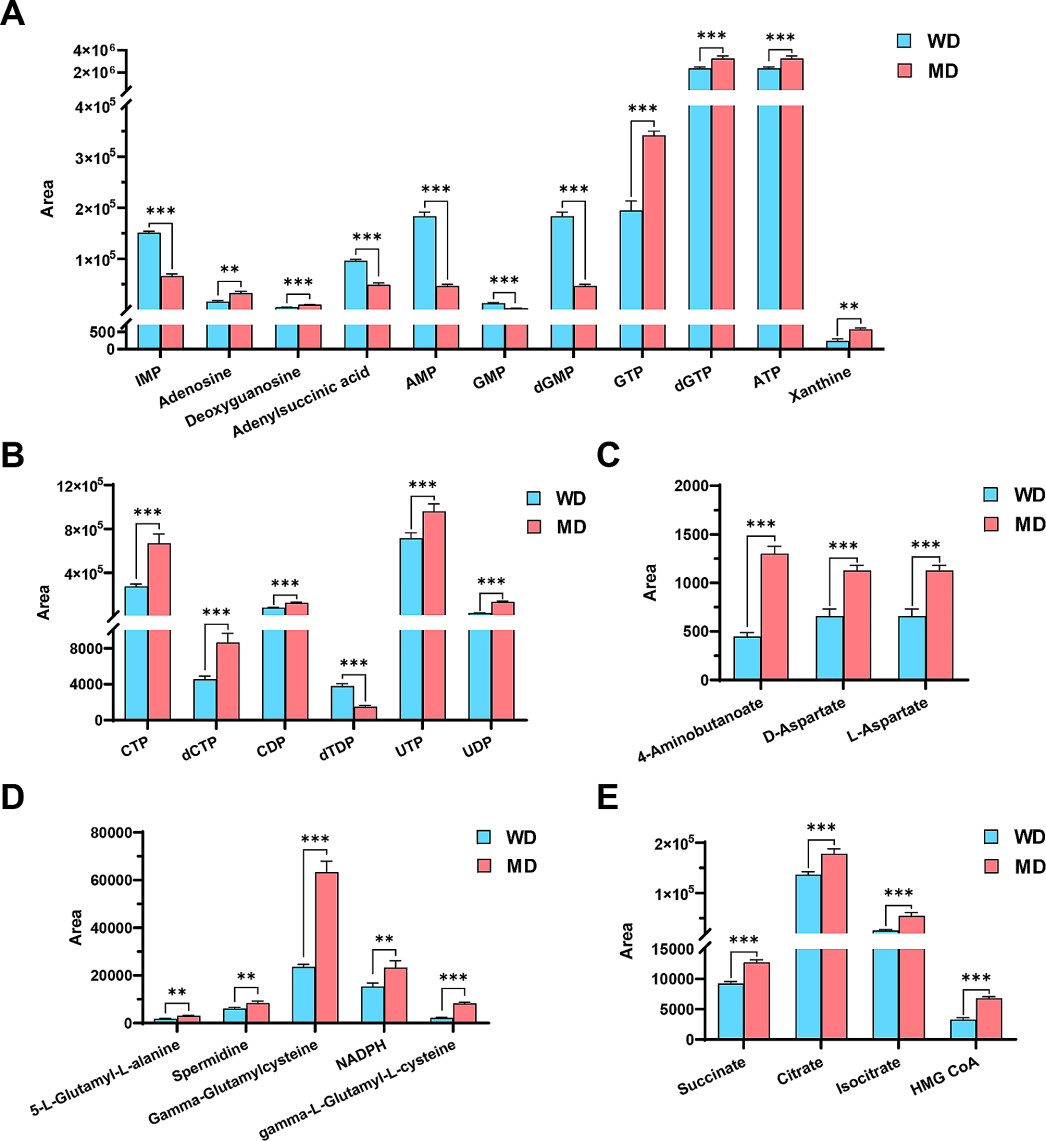
Expression profile of differential metabolites between WT and MF^+/-^ sheep skeletal muscle satellite cells at DM2. (**A**) Differential metabolite expression profiles related to purine metabolism. (**B**) Differential metabolite expression profiles related to pyrimidine metabolism. (**C**) Differential metabolite expression profiles related to alanine, aspartate and glutamate metabolism. (**D**) Differential metabolite expression profiles related to glutathione metabolism. (**E**) Differential metabolite expression profiles related to butanoate metabolism and TCA cycle. WD: WT sheep myotube cells at DM2; MD: MF^+/−^ sheep myotube cells at DM2

Further differential metabolite expression profiling revealed that the levels of adenylsuccinic acid, IMP, AMP, GMP, and dGMP in purine metabolism pathway of MF^+/−^ sheep myotube cells were remarkably (*P* < 0.01) diminished compared to WT sheep mytobue cells, while the levels of adenosine, deoxyguanosine, guanosine triphosphate (GTP), deoxyguanosine triphosphate (dGTP), ATP, and xanthine were markedly (*P* < 0.01) increased (Fig. 8A). In the pyrimidine metabolic pathways, uridine triphosphate (UTP), UDP, CTP, dCTP and CDP were all dramatically (*P* < 0.001) enhanced in MF^+/−^ sheep myotube cells, except that dTDP significant (*P* < 0.001) decreased (Fig. 8B). The levels of 4-aminobutanoate, D-aspartate and L-aspartate of the alanine, aspartate and glutamate metabolic pathways were dramatically (*P* < 0.001) increased in MF^+/−^ sheep myotube cells compared with WT cells (Fig. 8C). And the levels of 5-L-glutamyl-L-alanine, spermine, γ-glutamylcysteine, nicotinamide adenine dinucleotide phosphate (NADPH) and γ-L-glutamyl-L-cysteine of the glutathione metabolic pathway were also remarkably (*P* < 0.01) increased (Fig. 8D). Furthermore, the levels of succinate, citrate, isocitrate and DL-3-hydroxy-3-methylglutaryl-CoA (HMG CoA) related to the butanoate metabolism and TCA cycle in MF^+/−^ sheep myotube cells were dramatically (*P* < 0.001) increased (Fig. 8E).

## Discussion

### Metabolic reprogramming during myogenic differentiation of MuSCs in sheep

Metabolic regulation of skeletal muscle at the level of entire muscle and single muscle fibers has attracted extensive attention in the last century [19–21]. However, the energy requirements and metabolic state of MuSCs during the activation, proliferation and myogenic differentiation remain poorly understood. As previously described, the proliferation of myoblasts clearly depend on glycolysis for energy requirement, and both oxidative phosphorylation activity and mitochondrial density increased after differentiation [22, 23], indicating that differentiated MuSCs predominantly dependent on oxidative phosphorylation for energy supply. In the process of cell proliferation, DNA replication produces two identical chromosomes, and a large amount of mRNA and rRNA are synthesized to guide protein synthesis to support cell growth and metabolism. Therefore, the demand for nucleotides in proliferating cells increases. In fact, in the entire cellular metabolic network, the biosynthesis of nucleotides such as ribose, purine, and pyrimidine requires carbon, nitrogen, and energy sources from a variety of metabolic pathways, including the electron transport chains, one-carbon unit cycle, pentose phosphate pathway, TCA cycle, and various amino acid metabolisms. In this study, the metabolic pathways of purine, pyrimidine, alanine, glutamate and aspartate were weakened during sheep MuSCs myogenic differentiation, which may be the results of cell cycle exit caused by myogenic differentiation, and reduced cellular demand for DNA replication and cell proliferation. These results suggest that above metabolic pathways are more active during myoblasts proliferation, and that they are essential for maintaining the proliferation of sheep MuSCs. Notably, fatty acids such as arachidonic acid, prostaglandins and diglycerides can serve as important second messengers, but it remains unclear what role these signaling molecules play in the various states of skeletal muscle cells [24]. In this study, unsaturated fatty acids, such as linoleic acid, palmitic acid, arachidonic acid, α-linolenic acid, and docosahexaenoic acid in myotube cells were significantly up-regulated compared to MuSCs. It is hypothesized that the enhanced biosynthesis of unsaturated fatty acids may be related to the need to synthesize more membrane lipids during myogenic differentiation and the accumulation of large amounts of lipid droplets in myotube cells.

Pantothenic acid, contributes approximately 66% of CoA in skeletal muscle, is the main substrate of pantothenate kinase, which is the rate limiting progress of CoA biosynthesis [19]. In skeletal muscle energy metabolism, CoA replenishes the lipid acyl-CoA pool in mitochondria through the carnitine shuttle system. The lipid acyl CoA is involved in the formation of carnitine and acyl-CoA mediated by carnitine palmitoyltransferase 2 (CPT2), and is used for β-oxidation of mitochondrial fatty acid and pyruvate oxidative decarboxylation to produce acetyl-CoA, which ultimately becomes a pivotal substrate for α-ketoglutarate in TCA cycle [25]. More crucially, acyl-CoA synthetases selectively switch key fuels from glucose to fatty acids during active [26]. Metabolomic analysis of human primary MuSCs and myotube cells revealed that arginine, valine, and D-pantothenic acid were dramatically up-regulated in proliferating myoblasts, whereas oxidized glutathione, adenosine and glycerophosphocholine were markedly up-regulated in myotube cells, and the pantothenic acid metabolic pathway and CoA biosynthesis were weakened during myogenic differentiation compared to proliferating myoblasts [25]. In this study, we found that dephospho-CoA, PAP and P2A in the pantothenate and CoA biosynthesis pathways were dramatically down-regulated in differentiated sheep myotube cells, suggesting that the pantothenic acid and CoA biosynthesis pathways were also attenuated during myogenic differentiation. Furthermore, the high levels of fructose-6-phosphate and glucose-1-phosphate in the amino sugar and nucleotide sugar metabolic pathways of differentiated sheep myotube cells suggest that the glycolysis pathway may be inhibited, which is consistent with a shift from glycolysis-dependent to oxidative phosphorylation-dependent energy requirements during myogenic differentiation. In a word, the differential metabolites we identified in sheep MuSCs and myotube cells can serve as biomarkers of different cellular states, which has potential guidance for the proliferation and myogenic differentiation of MuSCs.

### Effect of MSTN and FGF5 dual-gene editing on the metabolic level of MuSCs and myotube cells in sheep

As previously mentioned, the demand for nucleotides in proliferating cells increases. Although the levels of alanine, aspartate, glutamate and purine metabolic pathway-related metabolites were decreased in MF^+/−^ sheep MuSCs, there were no significant changes in various purine and pyrimidine nucleotides except IMP. In addition, the levels of ribose-5-phosphate and ribose-1-phosphate that involved in the pentose phosphate pathway, and the interconversion of pentose and glucuronate were significantly elevated in MF^+/−^ sheep MuSCs. Among them, ribose-5-phosphate and ribose-1-phosphate are key molecules in nucleotide metabolism, ribose-5-phosphate can promote DNA synthesis and cell proliferation, while ribose-1-phosphate is involved in RNA and protein synthesis and promotes cell proliferation. These results further support our previous findings that *MSTN* and *FGF5* double gene editing promotes the proliferation of sheep MuSCs (Data Not Published). Oleic acids and linoleic inhibit the proliferation of mesenchymal stem cells [27], and the inhibition of oleic acid synthesis rate limiting enzyme salvages proliferative damage of the adult neurogenic niche in Alzheimer’s disease mice [28]. In current study, the levels of unsaturated fatty acids were significantly reduced in MF^+/−^ sheep MuSCs compared to WT cells, these results was contrary to the metabolic levels in sheep myotube cells, suggesting that MF^+/−^ sheep MuSCs may be in a more active proliferative state.

Triacylglycerols in lipid droplets usually contain unsaturated fatty acids, which are converted to triacylglycerols by uptake into the lipid droplets, and stored in cells when cell absorbs excess energy. The lipid droplets can facilitate myoblasts migration and fusion to form multinucleated myotubes by accelerating the remodeling of actin filaments [29]. Here, we observed that the levels of unsaturated fatty acid biosynthesis-related metabolites were dramatically diminished in MF^+/−^ sheep MuSCs, and the number of lipid droplets per satellite cell was also significantly decreased, suggesting that the limited biosynthesis of unsaturated fatty acids in MF^+/−^ sheep MuSCs may affect lipid droplet formation, thereby reducing cellular energy uptake and inhibiting myogenic differentiation. These results provide new evidence that *MSTN* and *FGF5* dual-gene editing inhibits myogenic differentiation.

Compared to WT sheep myotube cells, enhanced metabolic pathways for purine, pyrimidine, alanine, aspartate and glutamate in MF^+/−^ sheep myotube cells, suggesting that the demand for nucleotides in MF^+/−^ myotube cells remains higher, which also corroborates the lower myogenic differentiation capacity of MF^+/−^ sheep MuSCs. Interestingly, the levels of both substrates and products in the pathways of AMP and GMP synthesized by IMP were significantly reduced in MF^+/−^ sheep myotube cells. Whereas the levels of metabolites such as ATP and GTP that produced by twice phosphorylation of AMP and GMP under kinase catalysis were significantly increased, suggesting that *MSTN* and *FGF5* dual-gene editing may regulate the *de novo* synthesis of purine nucleotides in sheep myotubes. Citric acid is the main source of cytoplasmic acetyl-CoA for the biosynthesis of fatty acid and cholesterol, the pyruvate dehydrogenase complex can effectively oxidize pyruvate to produce acetyl-CoA and CO_2_ [30]. Acetyl CoA provides a carbon source for lipid biosynthesis by condensing with oxaloacetate to form citric acid, which is transferred to the cytoplasm for further metabolism [30]. The TCA cycle provides acetyl-CoA for acetylation and adipogenesis. In addition, the TCA cycle is activated during the early initiation stage of embryonic stem cell-specific differentiation [31]. Here, the levels of succinate, citrate and isocitrate, which are the intermediates of the TCA cycle, were significantly elevated in MF^+/−^ sheep myotube cells, suggesting that *MSTN* and *FGF5* dual-gene editing may promote the TCA cycle pathway in myotube cells and inhibit the level of glycolysis, thereby resulting in cells unable to obtain sufficient energy for myogenic differentiation. However, it is necessary to mention that more experiments are required to confirm in greater depth the influence of *MSTN* and *FGF5* dual-gene editing on TCA cycle, oxidative phosphorylation and glycolysis.

## Conclusion

Metabolic reprogramming occurs in myogenic differentiation of sheep MuSCs. In this process, the pathways of nucleotide metabolism, pantothenate and CoA biosynthesis were weakened, while unsaturated fatty acids biosynthesis was strengthened. The differential metabolites identified in sheep MuSCs and myotube cells can be characterized as biomarkers of different cellular states. The *MSTN* and *FGF5* dual-gene editing mainly inhibited nucleotide metabolism and unsaturated fatty acids biosynthesis in sheep MuSCs, promoted the pentose phosphate pathway, and the interconversion of pentose and glucuronate. The *MSTN* and *FGF5* dual-gene editing also mainly resulted in the inhibition of nucleotide metabolism and TCA cycle pathway in differentiated myotube cells.

### Electronic supplementary material

Below is the link to the electronic supplementary material.

Supplementary Material 1

Supplementary Material 2

Supplementary Material 3

Supplementary Material 4

Supplementary Material 5

## Data Availability

The data reported in this paper have been deposited in the OMIX, China National Center for Bioinformation / Beijing Institute of Genomics, Chinese Academy of Sciences (https://ngdc.cncb.ac.cn/omix: accession no. OMIX006516).

## Abbreviations

MuSCs: Skeletal muscle satellite cells
MSTN: Myostatin
FGF5: Fibroblast growth factor 5
GM: Growth medium
DM: Differentiation medium
WG: WT sheep MuSCs at GM
WD: WT sheep myotube cells at DM2
MG: MF^+/−^ sheep MuSCs at GM
MD: MF^+/−^ sheep myotube cells at DM2
OPLS-DA: Orthogonal partial least squares discriminant analysis
VIP: Variable important in projection
AMP: Adenosine monophosphate
IMP: Inosine monophosphate
GMP: Guanosine monophosphate
ADP: Adenosine diphosphate
GDP: Guanosine diphosphate
dGMP: Deoxyguanosine monophosphate
dATP: Deoxyadenosine triphosphate
dGDP: Deoxyguanosine diphosphate
UDP: Uridine diphosphate
UMP: Uridine monophosphate
CDP: Cytidine diphosphate
CMP: Cytidine monophosphate
CTP: Cytidine triphosphate
dCTP: Deoxycytidine triphosphate
dTDP: Deoxythymidine diphosphate
PAP: Adenosine 3’,5’-diphosphate
P2A: Adenosine 3’,5’-bisphosphate
GTP: Guanosine triphosphate
dGTP: Deoxyguanosine triphosphate
UTP: Uridine triphosphate
NADPH: Nicotinamide adenine dinucleotide phosphate
HMG-CoA: DL-3-hydroxy-3-methylglutaryl-CoA
LDs: Lipid droplets
